# Supplementary material for: Humoral and Cell-Mediated Immunity Against SARS-CoV-2 in Healthcare Personnel Who Received Multiple mRNA Vaccines: A 4-Year Observational Study
Source: Infect Dis Rep. 2025 Apr 29;17(3):42. doi: 10.3390/idr17030042 (PMC12101388; doi:10.3390/idr17030042)
Supplement: Supplementary file 1 [file idr-17-00042-s001.zip › idr-3569516-supplementary.pdf]

Figure S1

Characteristics of the subjects in this study. Age at the recruitment, sex, numbers of mRNA COVID-19 vaccine, and the timing of blood sampling, vaccination, and infection to SARS-CoV-2.

Age at the Apr/2021	Sex	Number of vaccination	SARS-CoV-2 infection (infected strains)	Vaccination [1]	Vaccination [2]	Blood sampling [A] April 2021	Blood sampling [B] September 2021	Vaccination [3]	Blood sampling [C] January 2022	Vaccination [4]	Vaccination [5] BA.1 bivalent	Vaccination [6] BA.4/5 bivalent	Infected Omicron	Blood sampling [D] July 2023	Vaccination [7] XBB.1.5	Infected Omicron XBB	Blood sampling [E] January 2024
40	Female	3	Omicron XBB	▲	▲	■	■	▲	■							★	■
37	Male	7	Omicron XBB	▲	▲	■	■	▲	■	▲	▲	▲		■	▲	★	■
46	Female	5	Omicron XBB	▲	▲	■	■	▲	■	▲		▲		■		★	■
34	Male	6	Omicron XBB	▲	▲	■	■	▲	■	▲	▲	▲		■		★	■
59	Female	7	Omicron XBB	▲	▲	■	■	▲	■	▲	▲	▲			▲	★	■
54	Female	6	Omicron XBB	▲	▲	■	■	▲	■	▲	▲	▲		■		★	■
61	Male	7	Omicron XBB	▲	▲	■	■	▲	■	▲	▲	▲		■	▲	★	■
43	Female	6	Omicron XBB	▲	▲	■	■	▲	■	▲	▲	▲		■	▲	★	■
57	Female	7	Omicron XBB	▲	▲	■	■	▲	■	▲	▲	▲		■	▲	★	■
36	Female	7	Omicron	▲	▲	■	■	▲	■	▲	▲	▲	★	■	▲		■
46	Female	5	Omicron	▲	▲	■	■	▲	■	▲	▲		★	■			■
42	Male	7	Omicron	▲	▲	■	■	▲		▲	▲	▲	★	■	▲		■
50	Female	5	Omicron	▲	▲	■	■	▲	■	▲	▲		★				■
47	Female	6	Omicron	▲	▲	■	■	▲	■	▲	▲		★	■	▲		■
56	Female	6	Omicron	▲	▲	■	■	▲	■	▲		▲	★	■	▲		■
51	Female	5	Omicron	▲	▲	■	■	▲	■	▲	▲		★				■
47	Female	3	Omicron	▲	▲	■	■	▲	■				★				■
47	Male	6	Omicron	▲	▲	■	■	▲	■	▲	▲	▲	★	■			■
48	Female	5	Non-infected	▲	▲	■	■	▲	■		▲			■	▲		■
49	Female	7	Non-infected	▲	▲	■	■	▲	■	▲	▲	▲			▲		■
61	Male	7	Non-infected	▲	▲	■	■	▲	■	▲	▲	▲		■	▲		■
39	Female	4	Non-infected	▲	▲	■	■	▲	■		▲						■
48	Female	7	Non-infected	▲	▲	■	■	▲	■	▲	▲	▲			▲		■
52	Female	7	Non-infected	▲	▲	■	■	▲	■	▲	▲	▲		■	▲		■
56	Male	7	Non-infected	▲	▲	■	■	▲	■	▲	▲	▲			▲		■
34	Male	7	Non-infected	▲	▲	■	■	▲	■	▲	▲	▲		■	▲		■
43	Female	7	Non-infected	▲	▲	■	■	▲	■	▲	▲	▲		■	▲		■
42	Male	6	Non-infected	▲	▲	■	■	▲	■		▲	▲		■	▲		■
33	Female	6	Non-infected	▲	▲	■	■	▲	■	▲	▲			■			■
45	Male	5	Non-infected	▲	▲	■	■	▲	■		▲	▲		■			■
40	Female	3	Non-infected	▲	▲	■	■	▲	■					■			■
48	Female	7	Non-infected	▲	▲	■	■	▲	■	▲	▲	▲		■	▲		■
